# Evidence for the Role of State-Level Economic Policy in HIV Risk Reduction: State Earned Income Tax Credit Generosity and HIV Risk Behavior Among Single Mothers

**DOI:** 10.1007/s10461-022-03754-x

**Published:** 2022-07-01

**Authors:** Kimberly Danae Cauley Narain, Nina Harawa

**Affiliations:** 1 Division of General Internal Medicine and Health Services Research, Department of Medicine, David Geffen School of Medicine at UCLA (DGSOM), University of California, Los Angeles, Los Angeles, CA USA; 2 Center for Health Advancement, Fielding School of Public Health, University of California Los Angeles, Los Angeles, CA USA; 3 Center for the Study of Racism, Social Justice, and Health Los Angeles, Los Angeles, CA USA; 4 Department of Psychiatry, Charles R. Drew University of Medicine and Science (CDU), Los Angeles, CA USA

**Keywords:** HIV, Risk-taking, Poverty, Health equity, Income

## Abstract

We investigated the impact of State-level Earned Income Tax Credit (SEITC) generosity on HIV risk behavior among single mothers with low education. We merged individual-level data from the Behavioral Risk Factor Surveillance System (2002–2018) with state-level data from the University of Kentucky Center for Poverty Research and conducted a multi-state, multi-year difference-in-differences (DID) analysis. We found that a refundable SEITC ≥ 10% of the Federal Earned Income Tax Credit was associated with 21% relative risk reduction in reporting any high-risk behavior for HIV in the last year, relative to no SEITC. We also found that a 10-percentage point increase in SEITC generosity was associated with 38% relative risk reduction in reporting any high-risk HIV behavior in the last year. SEITC policy may be an important strategy to reduce the burden of HIV infections among women with low socioeconomic status, particularly single mothers.

## Resumen

Investigamos el impacto de la generosidad del Crédito Federal Tributario por Ingreso de Trabajo a nivel estatal (SEITC) sobre el comportamiento de riesgo al VIH entre madres solteras con baja educación. Unimos los datos a nivel individual del Sistema de Vigilancia a Factores de Riesgo de Comportamiento (2002–2018) con los datos a nivel estatal del Centro de Investigación de la Pobreza de la Universidad de Kentucky, y conducimos un análisis de diferencia-en-diferencia (DID) multi-estado y multi-año. Encontramos que un reembolso SEITC ≥ 10% del Crédito Federal Tributario por Ingreso de Trabajo estaba asociado con una reducción relativa de riesgo de 21% en reportar cualquier comportamiento de riesgo alto al VIH en el último año, relativo a ningún SEITC. También encontramos que un aumento de punto porcentual de 10 en la generosidad SEITC estaba asociado con una reducción relativa de riesgo de 38% en reportar cualquier comportamiento de riesgo alto al VIH en el último año. La póliza SEITC puede ser una estrategia importante para reducir la carga de infecciones al VIH entre mujeres con bajo nivel socioeconómico, particularmente entre madres solteras.

## Introduction

The relationship between poverty and sexually transmitted infections (STIs) such as HIV is well-documented [1, 2]. For females in all age groups, those who lived in census tracts where the median household income was less than $36,000 a year experienced the highest rates of HIV diagnoses, according to 2013 analyses conducted by Centers for Disease Control and Prevention [3]. Poverty, low-wage jobs, income inequality, and other economic structural factors may spread STIs by creating high-risk partner pools, facilitating transactional sex, and undermining women’s sexual agency [4]. Loosier et al. found that relative to women who did not report food insecurity, women reporting food insecurity, a marker of economic vulnerability, had a 63% higher risk of reporting at least one of six behaviors included in a sexually transmitted infection risk indicator [(1) took money or drugs for sex in the past year; (2) had sex with male injection drug user in the past year; (3) chlamydia diagnosis in the past year; (4) gonorrhea diagnosis in the past year; (5) sex with an HIV-positive partner; and (6) sex with a non-monogamous partner] [5]. While a number of studies have investigated the implications of income support for sexual risk behavior among women in low and middle income countries, few studies have explored the extent to which income support may reduce participation in high-risk sexual behaviors among women in the United States context [6, 7]. Ibragimov et al. conducted an ecological study exploring the relationship between state-level minimum wage and STI rates and found that a $1 increase in the price-adjusted minimum wage was associated with a 19.7% and 8.5% decrease in syphilis and gonorrhea rates among women, respectively [4]. The findings of this study suggest that state-level policy may be an important strategy to reduce the risk of contracting HIV and other STIs among at-risk women.

The Federal Earned Income Tax Credit (FEITC) is a refundable tax credit for low-income workers that became law under President Gerald Ford in 1975. People do not have to owe taxes to receive it, but they must file an income tax return, have earned income, and meet income qualifications. The credit provides a subsidy as a percentage of income, effectively increasing the wages of the working poor [8]. The average annual FEITC has grown from $200 in 1975 to $2411 in 2020. Twenty-eight states and the District of Columbia have their own earned income tax credit programs, which provide additional credits on top of the federal one [9].

Between 1975 and 2016, the number of filers claiming the FEITC grew from 6.2 to 27.0 million. In 2015, about 70% of the FEITC filers had an adjusted gross income of $25,000 or less and the greatest earnings increases occurred among families with incomes of 75–100% of the poverty level [9]. During this same year, 97% of the benefits went to families with children and about 52% of the filers had a high school degree or less. With respect to the racial/ethnic breakdown of filers, slightly less than 50% were Non-Hispanic White, while 24% were Hispanic, and 19% were Non-Hispanic Black [9].

In 2016, the FEITC was estimated to lift 6.5 million people out of poverty and to reduce the severity of poverty for an additional 21.2 million families. This additional income is typically used by recipients to cover basic necessities, pay bills, reduce debt, make large purchases such as cars, pay rent, or make a security deposit for a rental unit [9]. A number of studies have linked FEITC laws to peri-natal health outcomes, mental health and risk behaviors among single mothers [10–13]. Given the well-established relationship between Earned Income Tax Credit laws and poverty reduction among single mothers, it is quite plausible that more generous state earned income tax credit laws may be associated with less engagement in high-risk behavior for HIV and other STIs.

We extend the literature examining the effects of earned income tax credit laws among single mothers by using Behavior Risk Factor Surveillance System (BRFSS) data and a quasi-experimental study design to examine the association between state earned income tax credit law generosity and HIV risk behaviors among single mothers with low education.

## Methods

To examine associations between state earned income tax credit law generosity and HIV risk behaviors among single mothers of minor children with low education, this study analyzes data from the BRFSS and state-level data from other sources covering the period from 2002 to 2018. We selected data from 2002 on because the question we used to construct our primary outcome has only been included in BRFSS core module questions since 2002. Prior to 2002, the question would only be asked of respondents residing in states that selected a separate module. We exclude 2007 BRFSS data from the analyses due to substantive changes in the wording of the question used to assess our primary outcomes during this particular year. Additionally, our data source for our primary predictor only included data through 2018. The BRFSS is conducted by state health departments, with technical and methodological assistance provided by the Centers for Disease Control and Prevention (CDC). This study sample includes single mothers, between the ages of 18 and 55, with minor children. To focus on low-skill workers, the sample was limited to those individuals who reported educational attainment of high school (or General Educational Development test) or lower. A dataset was constructed consisting of pooled cross-sectional observations at the individual level, combined with data on state earned income tax credit laws and several other state-level policy and labor force characteristics that vary over time and across states. These ecological data were obtained from the University of Kentucky Center for Poverty Research.

Our primary outcome is a composite HIV risk measure included in the BRFSS that was created based on input from the Division of HIV/AIDS Prevention at the CDC [14]. This variable assesses the presence of HIV risk behaviors/factors in the last year such as intravenous drug use, treatment for a sexually transmitted disease, exchange of money or drugs in exchange for sex and risky sexual practices (i.e., anal sex without a condom, having four or more sexual partners). The question has undergone some slight changes over time (Table 1). This HIV risk indicator is coded as “1” if there is an affirmative response for any of the behaviors listed in the question and “0” if none were endorsed.

**Table 1:** BRFSS HIV risk questions over time

2002–2012	I’m going to read you a list. When I’m done, please tell me if any of the situations apply to you. You don't need to tell me which one. *You have used intravenous drugs in the past year. You have been treated for a sexually transmitted or venereal disease in the past year. You have given or received money or drugs in exchange for sex in the past year. You had anal sex without a condom in the past year*
2016	I am going to read you a list. When I am done, please tell me if any of the situations apply to you. You do not need to tell me which one. *You have used intravenous drugs in the past year. You have been treated for a sexually transmitted or venereal disease in the past year. You have given or received money or drugs in exchange for sex in the past year. You had anal sex without a condom in the past year. You had four or more sex partners in the past year*
2017–2018	I am going to read you a list. When I am done, please tell me if any of the situations apply to you. You do not need to tell me which one. *You have injected any drug other than those prescribed for you in the past year. You have been treated for a sexually transmitted disease or STD in the past year. You have given or received money or drugs in exchange for sex in the past year*

To capture state earned income tax credit law generosity, we follow the approach used by Komro et al. and Markowitz et al. by creating five indicators based on the value of the credit as a percentage of the FEITC and refundability status: no state earned income tax credit (reference group for each variable), nonrefundable/< 10% FEITC, refundable/< 10% FEITC, nonrefundable/≥ 10% FEITC, and refundable/≥ 10% FEITC [10, 13]. The ≥ 10% FEITC reflects the 75th percentile of state earned income tax credit generosity for our study population. The “refundable” designation is important to consider in variable construction given that non-refundable tax credits are less available to this low-earning population that typically does not carry substantial tax liability [15]. We use a 1-year lagged version of the state earned income tax credit variable to reflect the fact that the question on HIV risk behavior references behavior over the prior year. We also construct a continuous version of the 1-year lagged state earned income tax credit variable based off of the %FEITC level, in an effort to assess for a dose–response relationship between state earned income tax credit generosity and HIV risk behavior. States without credits and those with non-refundable tax credits in a given year are assigned a “0” value for that particular year. States with non-refundable tax credits are assigned a “0” value because individuals without a high school education are less likely to have tax liability, reducing the likelihood that they will actually receive a refund [15].

The most salient study design challenge for evaluating the impact of state earned income tax credit generosity on HIV risk behavior is separating out this impact from those effects due to consistent state-specific characteristics as well as changes in the economy and temporally proximal changes in the policies of other means-tested programs that could potentially influence HIV risk behavior. In order to isolate the effect of changes in state earned income tax credit generosity from both consistent state-specific characteristics and other secular trends in the circumstances of single mothers, a multi-state, multi-year difference-in-differences (DID) study design was used [16]. A key assumption underlying the validity of the DID study design is that in the absence of state earned income tax credit generosity changes, the secular time trends for HIV risk behavior among single mothers in treatment states and comparison states would not be changing differentially [16].

Our equation is as follows:

$${\text{HIV}}\_{\text{Risk}}\_{\text{Behavior}}_{{{\text{ijt}}}} = \upbeta_{{1}} {\text{SEITC}}_{{{\text{j}}({\text{t}} - {1})}} + \upbeta_{{2}} {\text{X}}_{{{\text{ijt}}}} + \upbeta_{{3}} {\text{Z}}_{{{\text{jt}}}} + \updelta_{{\text{j}}} + \upgamma_{{({\text{t}})}} + \upvarepsilon_{{{\text{ijt}}}}$$

The dependent variable is reported “HIV risk behavior” for a single mother (i) in state (j) in year (t). The state earned income tax credit (SEITC) generosity variables are measures of the generosity of the 1-year lagged state earned income tax credit (described above) in effect in the state at the relevant time. The vector X contains maternal characteristics (age, race/ethnicity, number of minor children, health insurance coverage status and having a usual source of health care); Z represents state-level factors that are potentially associated both with the states’ decisions to implement a given state earned income tax credit law and also with HIV risk behavior (state 1-year lagged unemployment, state minimum wage, sum of the maximum monthly Temporary Assistance for Needy Families and food-stamp benefit amounts for a family of 3). δ_j_ represents state fixed-effects which attempts to control for time-invariant state-level differences in political culture, social or economic environment that are unmeasured but that would otherwise confound the analysis. γ_t_ represent year fixed-effects which attempts to controls for any national time trends that may be associated with changes to state earned income tax credit laws.

We calculated weighted proportions and means/standard deviations for our dichotomous and count variables, respectively. The unit of analysis was the person-year for all analyses. We applied a linear regression to our dichotomous outcome, also known as a linear probability model, which can be used when the sample size is large, since the normality distribution does not have to be assumed under these circumstances [17]. All models were weighted to account for complex survey design and non-response by using the sampling weights provided by the BRFSS. Additionally, robust standard errors clustered at the state level were used. A *p*-value cut off of ≤ 0.05 was used to determine statistical significance. We employed these adjustments through the SVY command with the linearized option in Stata version 13 (StataCorp LP, College Station, TX). All monetary values were adjusted for inflation to 2018 dollars.

To assess for differences in pre-treatment secular time trends in HIV risk behaviors, across individuals in treatment and comparison states, we limit our data set to the pre-treatment time period for all states and run linear probability models regressing HIV risk behavior on the interaction of treatment status (e.g., state earned income tax credit) and a linear time trend, controlling for all of our other covariates [18]. A statistically significant coefficient for the treatment status/linear time trend interaction term, during the pre-treatment period would suggest differences in the pre-treatment secular time trends, across the treatment and comparison groups, a violation of DID study design assumption.

## Results

The descriptive statistics for our outcome, predictor and covariates can be found in Table 2. Between the first and the last year of the analysis, the number of states without an EITC declined from 36 to 23. The reported prevalence of any HIV risk behavior during the study period is 6.1%. The mean and standard deviation of the 1-year lagged state earned income tax credit is 8.9% and 0.1% of the FEITC, respectively. The state earned income tax credit ranges from 0 to 85% of the FEITC. The results of our model assessing for differences in pre-treatment secular time trends (n = 122,314) did not suggest different baseline trends for HIV risk behavior across our treatment and comparison groups (β = − 0.03; t = − 0.46; 95% CI − 0.17 to 0.10) (not shown). Our adjusted analyses of state earned income tax credit generosity can be found in Table 3. We find that having a refundable credit $\ge$ 10% of the FEITC is associated with a 1.3%-point reduction (β = − 1.3; t = − 2.18, 95% CI − 2.5 to − 0.13) in reporting any high-risk HIV behavior, relative to no state earned income tax credit, controlling for other factors. Non-refundable credits and less generous credits did not show statistically significant associations with HIV risk behavior. Using the continuous version of the state earned income tax credit variable, we find that a 10%-point increase in state earned income tax credit generosity is associated with a 2.3%-point reduction in HIV risk behavior (β = − 2.3; t = − 2.10, 95% CI − 4.4 to − 0.14).

**Table 2:** Descriptive statistics for single mothers of minor children with low education between the ages of 18–55 responding to the Behavior Risk Factor Surveillance System (2002–2018)

	Mean (SD) or %
BRSSS HIV risk behavior	6.1% (0.1%)
State-level variables	
1-year lagged state earned income tax credit	8.9% (0.1)
1-year lagged unemployment	6.5% (0.1)
Minimum wage	$8.08/h (0.0)
Maximum monthly welfare/food stamp benefit (family of 3)	$1031.36 (0.6)
Individual-level variables	
Age	33.4 (0.4)
Non-Hispanic Black	13.7%
Hispanic	35.2%
Non-Hispanic White	54.8%
Number of minor children	2.1 (0.0)
No health insurance	30.4%
No usual source of care	30.1%

The data source is BRFSS (2002–2018 panels). All proportions and means/standard deviations are survey-weighted and calculated using the “svy” function with weights provided by BRFSS

*SD* standard deviation

**Table 3:** The association of state earned income tax credit generosity with HIV risk behavior among single mothers of minor children with low education

SEITC type	HIV risk behavior	T score	*p* value
≥ 10% FEITC/refundable	− 1.3	− 2.18	0.03
≥ 10% FEITC/non-refundable	0.8	0.95	0.34
< 10% FEITC/refundable	− 0.1	− 0.31	0.76
< 10% FEITC/non-refundable	− 1.0	− 1.36	0.17
Continuous SEITC^a^	− 2.3	− 2.10	0.04

The data source is BRFSS (2002–2018 panels). Linear Probability Models were used to estimate the relationship between the 1-year lagged State Earned Income Tax Credit (SEITC) profile and HIV risk behavior. The point estimates reflect predictive margins (percentage point change in HIV-risk behavior associated with a given SEITC law, relative to no SEITC law). All models controlled for 1-year lagged unemployment, minimum wage, welfare generosity (cash transfers and food stamp benefits), individual-level factors (age, race/ethnicity, number of minor children, health insurance coverage, usual source of care), and included fixed effects for state and year. Standard errors were robust and clustered at the state-level. All monetary values were adjusted for inflation. Estimates are adjusted for non-response and complex survey design using sampling weights. N = 207, 238

^a^The continuous version of SEITC replaces non-refundable tax credits with a value of “0”

## Discussion

This study used BRFSS data and a quasi-experimental study design to explore the relationships between state earned income tax credit generosity and HIV risk behavior, among single mothers of minor children with low education. Controlling for several potential confounders, we found that a refundable state earned income tax credit $$\ge$$ 10% of FEITC was negatively associated with reporting any HIV risk behavior. This relationship was also observed using a continuous version of the state earned income tax credit variable; however, no association was found for non-refundable state earned income tax credit laws. Inability to detect a statistically significant relationship between HIV risk behavior and non-refundable state earned income tax credit laws may reflect a decreased likelihood of accessing these credits due to the lower average level of tax liability among this population [15]. Likewise, a refundable credit less than 10% of the FEITC (FEITC = $6431 for a family with 3 or more children in 2018) may not be large enough to have a meaningful impact on HIV risk behavior for low-income single mothers [19].

This is the first study to examine the relationship between state earned income tax credit laws and HIV risk behavior. The finding of a negative association between state earned income tax credit generosity and a composite measure of HIV risk behavior is particularly important given that the population observed in this study is at high risk for contracting HIV [3]. The finding of a 1.3%-point reduction in reporting any HIV risk behavior, associated with the most generous state earned income tax credit, relative to no credit, reflects a 21% relative risk reduction in HIV risk behavior. The analysis with the continuous measure of the state earned income tax credit suggest more than a 30% relative reduction in any HIV risk behavior associated with a 10%-point increase in the generosity of a refundable credit. According to a meta-analysis conducted by O’Connor et al. published in the *Annals of Internal Medicine*, this effect is on par with what would be expected for two or more hours of intensive HIV risk reduction counseling [20], an intervention that most low-income single mothers are unlikely to receive. Additionally, compared with at-risk men, individuals represented by the study population typically have less access to pre-exposure and post-exposure prophylaxis, interventions that have been integral in reversing the HIV trends among more affluent subsets of the population [21, 22]. Furthermore, it is worth noting that of the ten states with the highest HIV rates in 2018 (Florida, Hawaii, Georgia, Louisiana, Nevada, Maryland, Mississippi, Texas, South Carolina and New York), only four had any form of state earned income tax credit (Hawaii, Louisiana, South Carolina and New York), only two of the states had a refundable credit (Louisiana and New York) and only one of the states had a refundable credit > 10% of FEITC (New York) [23]. As such, there may be an important opportunity to influence the trajectory of HIV in this population through increased uptake of state earned income tax credit laws, changes in the way the laws are implemented and increases in benefit generosity. Lastly, the HIV risk behaviors reflected in our composite HIV risk variable are implicated in a number of other infections that may have long-term health sequela such as pelvic inflammatory disease, cervical cancer and liver cancer [24]. Consequently, the long-term health and healthcare costs implications of more generous state earned income tax credit laws may be sizable.

The results of this study must be interpreted in the context of several limitations. The study is observational and could be subject to residual confounding. The shortcomings of the BRFSS with respect to potential selection bias have been previously documented; it is worth noting that underrepresentation of individuals without landlines and those working atypical hours necessarily exclude individuals who may be disproportionately affected by state earned income tax credit laws [25]. A measurement limitation is the changes in the question used assess HIV risk behavior over time; however, we have no reason to suspect that these changes would have differentially impacted individuals in our treatment and comparison states, minimizing this issue as substantive source of bias in our results. Additionally, the results of this study may underestimate the impact of state earned income tax credit generosity on HIV risk behavior in two different ways. First, the study uses an intent-to-treat approach which classifies everyone in a state with a state earned income tax credit law as part of the treatment population; however, at least 20% of individuals may not have actually received this benefit [9]. While this intent-to-treat approach minimizes selection bias, it will bias findings towards the null. Second, the underestimation of the effect of state earned income tax credit laws may be attributable to reduced sensitivity of the HIV risk behavior question for detecting non-commercial forms of transactional sex such as staying with a partner longer than desired, starting a new sexual relationship or having sex with someone who is not a regular partner under the implicit assumption that sex will be exchanged for material support or other benefits [26].

Given the potential benefits and minimal downsides associated with more generous state earned income tax credit policies, these findings emphasize the potential benefits of increased uptake of generous policies. Traditionally, laws that encourage work have enjoyed bi-partisan support and have drawn less criticism from employers. So state earned income tax credit laws may prove more politically feasible to implement than other economic policies such as minimum wage increases. Additionally, increasing outreach to improve uptake among potentially eligible filers may also result in population health benefits. As it stands, only about 80% of potentially eligible filers access the federal earned income tax credit benefit; it is unknown how many access state earned income tax credit benefits [9]. It is likely that even fewer access the state earned income tax credit benefit. While not the focus of this study, future studies should consider exploring how federal income support initiatives, such as the increases in the child income tax credit under the American Rescue Plan, may have influenced HIV risk behaviors among single mothers with low education and others who may be at risk for HIV/STIs [27]. Such information may be useful for thinking about the return on investment associated with continuing these credits through the Build Back Better package of legislation or as a stand-alone policy [28].

## Data Availability

Data is publicly available at https://www.cdc.gov/brfss/.
